# Visual Field Abnormalities among Adolescent Boys with Hearing Impairments

**Published:** 2016

**Authors:** Masoud KHORRAMI-NEJAD, Javad HERAVIAN, Mohamad-Reza SEDAGHAT, Hamed MOMENI-MOGHADAM, Davood SOBHANI-RAD, Farshad ASKARIZADEH

**Affiliations:** 1 Refractive Errors Research Center, School of Paramedical Sciences, Mashhad University of Medical Sciences, Mashhad, Iran; 2 Department of Optometry, School of Paramedical Sciences, Mashhad University of Medical Sciences, Mashhad, Iran; 3 Cornea Research Center, Khatam‐Al‐Anbia Hospital, Mashhad University of Medical Sciences, Mashhad, Iran; 4 Department of Speech Therapy, School of Paramedical Sciences, Mashhad University of Medical Sciences, Mashhad, Iran

**Keywords:** Visual Field Abnormalities, Adolescent Boy, Hearing Impairments

## Abstract

The aim of this study was to compare the visual field (VF) categorizations (based on the severity of VF defects) between adolescent boys with hearing impairments and those with normal hearing. This cross-sectional study involved the evaluation of the VF of 64 adolescent boys with hearing impairments and 68 age-matched boys with normal hearing at high schools in Tehran, Iran, in 2013. All subjects had an intelligence quotient (IQ) > 70. The hearing impairments were classified based on severity and time of onset. Participants underwent a complete eye examination, and the VFs were investigated using automated perimetry with a Humphrey Visual Field Analyzer. This device was used to determine their foveal threshold (FT), mean deviation (MD), and Glaucoma Hemifield Test (GHT) results. Most (50%) of the boys with hearing impairments had profound hearing impairments. There was no significant between-group difference in age (P = 0.49) or IQ (P = 0.13). There was no between-group difference in the corrected distance visual acuity (P = 0.183). According to the FT, MD, and GHT results, the percentage of boys with abnormal VFs in the hearing impairment group was significantly greater than that in the normal hearing group: 40.6% vs. 22.1%, 59.4% vs. 19.1%, and 31.2% vs. 8.8%, respectively (P < 0.0001). The mean MD in the hearing impairment group was significantly worse than that in the normal hearing group (-0.79 ± 2.04 and -4.61 ± 6.52 dB, respectively, P < 0.0001), and the mean FT was also significantly worse (38.97 ± 1.66 vs. 35.30 ± 1.43 dB, respectively, P <0.0001). Moreover, there was a significant between-group difference in the GHT results (P < 0.0001). Thus, there were higher percentages of boys with VF abnormalities and higher mean MD, FT, and GHT results among those with hearing impairments compared to those with normal hearing. These findings emphasize the need for detailed VF assessments for patients with hearing impairments.

## INTRODUCION

Among children with hearing impairments, having a normal visual system is essential for developing efficient communication skills and for discovering and extracting information from the world (1). Notably, ocular and visual anomalies such as refractive errors, difficulty sustaining visual attention (2), and ocular motor abnormalities (3) are more likely to occur in children with hearing impairments compared to those with normal hearing and the general population. These findings emphasize the importance of early visual assessments for people with hearing loss in order to diagnose any vision-related impairment when they are young (1). The prevalence of birth defects that cause hearing impairments is 0.1–0.3% (4, 5), and > 20 million people in the United States are affected by hearing impairments (6). In individuals with profound hearing impairments, the detection of environmental changes and orienting of attention rely primarily on vision, and sensory deprivation is associated with crossmodal neuroplastic changes in the brain (7-9). Merabet and Pascual-Leone showed that auditory or visual deprivation can lead to the recruitment of the brain areas normally associated with the deprived sense for use by the spared senses; however, these changes can sometimes be maladaptive in light of potential of rehabilitative efforts to restore sensory function after it has been lost or fails to develop (10). Early detection and appropriate management of these adaptations and of defects such as visual field (VF) abnormalities can improve a patient’s vision-related quality of life (11, 12). VF loss is associated with a risk of other vision-related impairments (11, 12), and early detection of these impairments is also important.

Many researchers have evaluated the visual performance of patients with hearing impairments. For example, Scott et al. used functional magnetic resonance imaging to show that there was asymmetry in peripheral visual processing among individuals with hearing impairments (13), and Rothpletz et al. reported slower responses to peripheral visual targets among adults with hearing loss compared those with normal hearing (14). However, the majority of these studies have focused on the prevalence of visual abnormalities, and few studies have examined the VF and peripheral visual processing in individuals with hearing loss. This study was designed to investigate VF categorizations (based on the severity of VF defects) and their association with other visual parameters in adolescent boys with hearing impairments. In addition, the outcomes were compared to normative data from age-matched boys with normal hearing.

## MATERIALS AND METHODS

**Study Design and Ethics Approval**

The study is a cross-sectional study that was conducted from March to June 2013, and the study team consisted of an optometrist, an ophthalmologist, a speech therapist, an audiologist, and a psychologist. The ethics committee at Mashhad University of Medical Sciences approved the study protocol in 2012 (registration number: 900857), and the study was performed according to the tenets of the Declaration of Helsinki. After a verbal explanation of the aim of the study and the methods that would be used was provided to the potential subjects and their parents, written informed consent was obtained from all the boys included in the study and their parents. 

**Study Subjects**

The cases were male high school students with IQs > 70 who had hearing impairments, and the controls were male high school students with IQs > 70 and normal hearing. The two samples were randomly selected from the state high schools in Tehran, Iran (with the cases being recruited from high schools for deaf students). Personal information (including age and ethnicity) was documented. Subsequently, to ensure the accuracy of tests (15), the potential subjects completed an IQ test (the Wechsler Intelligence Scale for Children-Revised (WISC-R), which has a reliability and validity of 0.73 (16-19)) to determine which students met the eligibility threshold of > 70. 

We excluded boys with systemic diseases (such as diabetes), those who took certain medications and consumed special drugs, those who had ocular pathologies, those who had undergone previous eye surgery, and those who cooperated poorly during the examinations. 

As a result, the study sample consisted of 64 subjects in the hearing impairments group and 68 in the normal hearing group. Due to the correlation between the outcomes for paired eyes of single subjects (which can lead to underestimation of P values), the data of only one eye (the right eye) of each subject were included in the analysis.

**Audiometry**

The hearing impairments of the cases were investigated by the audiologist. In accordance with a study by Hollingsworth et al., the subjects were classified into different groups based on the severity of their hearing impairment (mild [20 – 40 dB], moderate [41 – 70 dB], severe [71 – 95 dB], and profound [> 95 dB] and time of onset (congenital and acquired) (3).

**Ocular and Visual Assessments**

All subjects underwent a full ophthalmic examination (including a biomicroscopic evaluation and ophthalmoscopy) to exclude the boys with ocular pathologies (including corneal or lenticular opacities). Refractive errors were measured objectively and the results were refined using a subjective refraction assessment. The corrected distance visual acuity (CDVA) was measured using a standard Snellen chart at a distance of 20 ft, and it was recorded in Logarithm of the Minimum Angle of Resolution (logMAR) units. VF was evaluated using a Humphrey 750*i* Visual Field Analyzer (Carl Zeiss Meditec AG, Jena, German). All the measurements obtained using this device were done so in a consistent manner, based on the manufacturer’s guidelines, and a representative of the manufacturer checked the calibration of the device before the measurements were taken. The central VF was assessed using the 30-2 Full Threshold program. This program tests 76 points within the central 30°, and it uses a 6°-spaced grid offset from the vertical and horizontal meridians. After the subject was prepared and the ambient lighting in the room was reduced, the 30-2 program was selected from the main menu. The subject was allowed to adapt to the luminance while entering their information. The non-test eye was occluded and the subject held the response button in their hand. Whenever necessary, trial lenses were placed in front of the test eye as close to the eye as possible without touching the eyelashes. 

After ensuring the validity of the results for each subject with respect to the reliability indices (i.e., fixation losses, false-negative error, and false-positive error), the following variables were recorded: foveal threshold (FT) in dB, mean deviation (MD) in dB, and glaucoma hemifield test (GHT) result. We interpreted the automated perimetry outcomes based on those of previous studies (20-22). The GHT results were classified as normal if they were “within normal limits” and abnormal if they were “outside normal limits” or “borderline” (20, 21)). In accordance with previous studies, abnormal VFs were categorized into three groups based on their severity: normal, mild, moderate, and severe (23, 24). After repeating the tests for the other eye, the results were printed out in a single-field analysis format.

**Statistical Analysis**

Statistical analysis was performed using Statistical Package for Social Sciences (SPSS) for Windows version 22 (IBM Inc., Chicago, IL, USA). Descriptive statistics (means, standard deviations, frequencies, and proportions) were calculated. The normality of the data was assessed using the Kolmogorov–Smirnov test, and parametric and nonparametric tests were applied accordingly. We used independent-samples t-tests and Fisher’s exact tests to determine whether there were significant between-group differences in the means and proportions, respectively. P values < 0.05 were considered statistically significant.

## RESULTS

There was no significant between-group difference in age (P = 0.49), with a mean age of 16 in both groups and a range of 14 – 18 years in both groups. There was also no significant between-group difference in IQ (P = 0.13), with a mean IQ in the hearing impairment and normal hearing group of 97.8 (range: 61 – 129) and 101.1 (range: 61 – 138), respectively. The groups were also well matched in terms of ethnicity. Table 1 shows the frequency of hearing impairments by severity and time of onset in the hearing impairment group. In terms of the severity of the hearing impairments in the hearing impairment group, none of the boys showed a mild impairment. If the moderate and severe impairments are categorized in one group and the profound impairments are categorized in another group, Fisher’s exact test indicated that there was no significant between-group difference in the time of onset of hearing loss (P = 0.78). The mean CDVA in the hearing impairment and control group was 0.03 ± 0.07 logMAR (range: 0.0 – 0.30 logMAR) and 0.01 ± 0.06 logMAR (range: 0.00 – 0.40 logMAR), respectively, with no significant between-group difference (P = 0.183). Furthermore, 86% and 96% of the boys with hearing impairments and those with normal hearing, respectively, had a CDVA of 0.00 logMAR or better. The frequency distribution of the different types of refractive errors in the two groups is presented in Table 2. The frequency distributions of MD, FT, and GHT classifications in the two groups are displayed in Table 3.

The frequency distribution in the two groups of the VF categorizations (including the distribution by the severity and time of onset of hearing impairment for those in the hearing impairment group) is displayed in Table 4.

**Table 1:** Severity of hearing impairments by time of onset in the hearing impairment group (n = 64)

**Severity**	**Moderate**	**Severe**	**Deep**	**Total**
**Congenital**	6 (9.4)	20 (31.2)	31 (48.4)	57 (89.1)
**Acquired**	-	6 (9.4)	1 (1.5)	7 (10.9)
**Total**	6 (9.4)	26 (40.6)	32 (50.0)	64 (100.0)

Data in table are presented and No. (%).

**Table 2:** Types of refractive error in the hearing impairment and control groups

**Type of RE**	**Hyperopia (≥ +1.00 D)**	**Myopia (≥ -0.50 D)**	**Astigmatism ** *
**Hearing Loss (n = 64)**	14 (21.9%)	5 (7.8%)	14 (21.9%)
**Control (n = 68)**	12 (17.6%)	9 (13.2%)	10 (14.7%)

* Astigmatism (Myopia/ Plano/ Hyperopia + Astigmatism ≥ 0.75)

**Table 3:** Foveal Threshold, Mean Deviation, and Glaucoma Hemifield Test classifications in the hearing impairment and control groups

**Test and Status**	**Hearing n (%)**	**Hearing loss n (%)**
**GHT**		
Normal	62 (91.2)	44 (68.8)
Abnormal	6 (8.8)	19 (31.2)
**MD**		
Normal	55 (80.9)	26 (40.6)
Abnormal	13 (19.1)	38 (59.4)
**FT**		
Normal	53 (77.9)	38 (59.4)
Abnormal	15 (22.1)	26 (40.6)

GHT: Glaucoma Hemifield Test, MD: Mean Deviation, FT: Foveal Threshold. There was a significant difference in the mean GHT result between the hearing impairment and normal hearing group (P < 0.0001).

The percentage of boys with normal VF and mild, moderate, and severe VF defects in subjects with congenital versus acquired hearing loss was 43.9% vs. 28.6%, 24.6% vs. 42.9%, 10.5% vs. 14.3%, and 21.1% vs. 14.3%, respectively. The percentage of boys with each of the three types of VF defect (based on the FT, MD, and GHT results) was significantly greater in the hearing impairment group compared to those in the normal hearing group: 40.6% vs. 22.1%, 59.4% vs. 19.1%, and 31.2% vs. 8.8%, respectively (P < 0.0001). The mean MD and FT in the two groups is presented in Table 5. There was a significant difference in the mean MD (P < 0.0001) and FT (P < 0.0001) between the two groups.

**Table 4:** Visual field categorization (based on the severity of defects) in the hearing impairment and control groups, and the visual field categorization by severity and time of onset of hearing loss for the boys in the hearing impairment group

**VF Status**	**Abnormal**						**Normal**
	**Moderate**		**Severe**		**Deep**		
	**Congenital**	**Acquired**	**Congenital**	**Acquired**	**Congenital**	**Acquired**	
**Normal**	4 (6.2)	-	10 (15.6)	2 (3.1)	11 (17.2)	-	48 (70.5)
**Abnormal**							
**Mild**	1 (1.6)	-	3 (4.7)	3 (4.7)	10 (15.6)	-	12 (17.6)
**Moderate**	1 (1.6)	-	2 (3.1)	-	3 (4.7)	1 (1.6)	6 (8.8)
**Severe**	-	-	5 (7.8)	1 (1.6)	7 (10.9)	-	2 (2.9)
**Total**	6 (9.4)	-	20 (31.2)	6 (9.4)	31 (48.4)	1 (1.6)	68 (100)

Data in table are presented as No. (%).

**Table 5:** Mean deviation and foveal threshold (dB) in the hearing impairment and control groups

	**Mean ± SD**	**Maximum**	**Minimum**
**Mean deviation**			
Hearing	-0.79 ± 2.04	2.44	-7.48
Hearing Loss	-4.61 ± 6.52	2.15	-26.95
**Foveal Threshold**			
Hearing	38.97 ± 1.66	43.00	35.00
Hearing Loss	35.30 ± 1.43	38.00	31.00

## DISCUSSION

Using a Humphrey Visual Field Analyzer and an automated perimetry method (which is the gold standard method for VF testing (22)), this study showed that VF abnormalities were more common in boys with hearing impairments compared to those with normal hearing. The percentage of boys with abnormal FT, MT, and GHT results among the boys with hearing impairments versus those with normal hearing was 40.6% vs. 22.1%, 59.4% vs. 19.1%, and 31.2% vs. 8.8%, respectively (P < 0.0001). However, the normality values for MD and FT in the two groups were significantly different. Earlier studies have shown that there is a high incidence of visual impairments (such as refractive errors, strabismus, and ocular pathologies) in the hearing-impaired population. However, none of these studies explored the type and severity of VF abnormalities. Dye et al. stated: “following early auditory deprivation, visual attention resources toward the periphery slowly get augmented to eventually result in a clear behavioral advantage by pre adolescence on a selective visual attention task” in their study on the use of a Useful Field of View test in individuals with hearing impairments (25).

In this study, 14% of the boys with hearing impairments had a CDVA > 0.00 logMAR. This finding is similar to that of a study by Arming et al., which showed that even after perfect correction of the eyes, 10.8% of the subjects did not have normal visual acuity (26). The most common types of refractive error in the boys with hearing impairments were hyperopia and astigmatism. The least common type was myopia (7.8%), which concurs with the results of a study by Mohindra et al., which showed that 5.8% of X had myopia (27). KhorramiNejad et al. reported that the percentage of boys with hearing loss who had any type of refractive error was 39.9%, and the percentage myopia in particular was 12.6% (28). The lower percentage of boys with myopia in our study may be attributable to the exclusion of boys with ocular pathologies, which did not occur in the study by KhorramiNejad et al. (28). The most common VF category (based on the severity of VF defects) in both groups was normal, and the least common category in both groups was moderate. The second most common category in the hearing impairment group was mild. Using an arcuate perimeter, Khandekar et al. assessed peripheral VFs in people with hearing loss, and they found that only one subject had a VF defect (but there was no information on the type and severity of this defect) (29). The significant differences between our study and this previous study may be attributable to differences in methodology and test sensitivity. Using a manual Goldmann perimeter, Buckley et al. showed that there was a significant increase in the size of the VF in people with hearing loss compared to those with normal hearing (30). This contrasts with our results, which indicate that the hearing impairment group had a higher percentage of FT abnormalities compared to the normal hearing group. However, there were differences in our study and the study by Buckley et al. in terms of the aims and methods. 

In another study, Codina et al. used a static perimetry technique that was specially designed for children and found that the response to environmental stimuli among children with hearing loss involved significantly shorter reaction times compared to those among children with normal hearing (31). In contrast to their special perimetry method for evaluating the response time to environmental stimuli, we studied VF global indices in boys with normal hearing and hearing impairments. In addition, Codina et al. studied the differences in the response time to environmental stimuli among children of different ages while we compared the VF abnormalities between students with hearing impairments and those with normal hearing.

## CONCLUSIONS

Despite the fact that individuals with hearing impairments are more dependent on a healthy VF in comparison to those with normal hearing, people with hearing loss have a considerable risk of having visual problems. A higher percentage of adolescent boys in the hearing impairment group had VF abnormalities compared to those in the normal hearing group. In addition, the mean MD, FT, and GHT results were worse in boys with hearing impairments compared to those with normal hearing. This indicates the importance of carrying out VF diagnostic tests among people with hearing impairments.
